# Understanding how geographic, demographic and treatment history impact health outcomes of patients with multi-drug-resistant tuberculosis in Pakistan, 2014–2017

**DOI:** 10.1017/S0950268820002307

**Published:** 2020-09-30

**Authors:** F. Iqbal, M. K. Defer, A. Latif, H. Hadi

**Affiliations:** 1 Harvard Medical School, Harvard University, Boston, USA; 2 School of Public Health and Health Systems, Faculty of Applied Health Sciences, University of Waterloo, Waterloo, Canada; 3 National Tuberculosis Control Program Pakistan, Islamabad, Pakistan; 4 Brigham and Women's Hospital, Boston, USA

## Abstract

Tuberculosis (TB) is one of the top 10 leading causes of morbidity and mortality worldwide [1]. In 2017, approximately 10 million people were infected with TB and 1.3 million patients faced mortality [1]. Patients with active TB can infect up to 10–15 people over a year. There is a greater risk of transmission in overcrowded areas with limited air ventilation including large family units, prisons and slums [1, 2]. Without proper diagnosis and treatment, roughly 45% of non-HIV positive TB patients face mortality [1]. With the help of global organizations and national TB treatment and control programmes, the global incidence of TB is declining by approximately 2% each year [1]. The World Health Organization (WHO) TB-strategy aims to end the TB epidemic and encourages partners to fund national TB programmes to improve diagnosis and treatment of TB. The goal is to ultimately decrease death rates by 90% and decrease incidence rates by 80% [1]. To achieve these goals, the decline in TB incidence needs to reach approximately 4–5% per year [1]. The WHO 2018 TB report identified multidrug resistant TB (MDR-TB) as the leading factor hindering that goal [1]. The incidence and spread of MDR-TB has drastically increased, where approximately 558 000 new cases of MDR-TB were diagnosed in 2017 causing more than 230 000 deaths globally [1]. MDR-TB is identified by resistance to the two most powerful anti-TB treatment drugs including isoniazid and rifampicin [3]. Patients with MDR-TB are required to start second-line anti-TB drugs (SLDs), which are limited, expensive, less effective and more toxic [1,2]. Therapy duration is one of the major limitations of second-line treatments, which may require up to two years of consistent use. Since TB affects mostly developing countries, long treatment durations and associated costs become a major challenge. In 2015, 15% of new TB cases were reported as MDR-TB, which drastically increased to 24% by 2017 [1]. Even with significant improvements in molecular tests and diagnostic methods, MDR-TB is still on the rise where the success rate of treatments is between 50 and 60% [1]. Additional characteristics including socioeconomic and sociocultural factors need to be considered when targeting and treating patients with MDR-TB.

## Introduction

Tuberculosis (TB) is one of the top 10 leading causes of morbidity and mortality worldwide [1]. In 2017, approximately 10 million people were infected with TB and 1.3 million patients faced mortality [1]. Patients with active TB can infect up to 10–15 people over a year. There is a greater risk of transmission in overcrowded areas with limited air ventilation including large family units, prisons and slums [1, 2]. Without proper diagnosis and treatment, roughly 45% of non-HIV positive TB patients face mortality [1]. With the help of global organizations and national TB treatment and control programmes, the global incidence of TB is declining by approximately 2% each year [1]. The World Health Organization (WHO) TB-strategy aims to end the TB epidemic and encourages partners to fund national TB programmes to improve diagnosis and treatment of TB. The goal is to ultimately decrease death rates by 90% and decrease incidence rates by 80% [1]. To achieve these goals, the decline in TB incidence needs to reach approximately 4–5% per year [1]. The WHO 2018 TB report identified multidrug resistant TB (MDR-TB) as the leading factor hindering that goal [1]. The incidence and spread of MDR-TB has drastically increased, where approximately 558 000 new cases of MDR-TB were diagnosed in 2017 causing more than 230 000 deaths globally [1]. MDR-TB is identified by resistance to the two most powerful anti-TB treatment drugs including isoniazid and rifampicin [3]. Patients with MDR-TB are required to start second-line anti-TB drugs (SLDs), which are limited, expensive, less effective and more toxic [1,2]. Therapy duration is one of the major limitations of second-line treatments, which may require up to two years of consistent use. Since TB affects mostly developing countries, long treatment durations and associated costs become a major challenge. In 2015, 15% of new TB cases were reported as MDR-TB, which drastically increased to 24% by 2017 [1]. Even with significant improvements in molecular tests and diagnostic methods, MDR-TB is still on the rise where the success rate of treatments is between 50 and 60% [1]. Additional characteristics including socioeconomic and sociocultural factors need to be considered when targeting and treating patients with MDR-TB.

Pakistan, with an approximate population of 197 million people is currently ranked fifth worldwide for the highest TB incidence [4]. According to the WHO Global Tuberculosis 2018 Report, Pakistan had approximately 525 000 new TB cases and an estimated 27 000 new drug-resistant TB cases annually [1]. Pakistan has the fourth highest incidence rate of MDR-TB. The national prevalence survey conducted in 2010–2011 suggests the prevalence of 270 sputum smear-positive patients per 100 000 people. In addition to this, the survey indicated a significant increase in the prevalence rate of bacteriologically confirmed TB in patients older than age 55 [1, 5]. In 2017, there were 27 000 cases of rifampicin-resistant TB patients, of which only 3475 were diagnosed and only 2081 were started on treatment [1]. Studies identify prior exposure to TB or MDR-TB, previous history with anti-TB medication, delayed diagnosis, inadequate drug regimens, limited follow-ups, age, gender, low socioeconomic status, limited education status and poor patient compliance as associated risk factors for MDR-TB [6–8]. The healthcare system in Pakistan includes both public and private healthcare facilities. At the time of the study, the MDR-TB treatment sites consisted of 21 public hospitals and 3 private hospitals, a total of 24 Programmatic Management of Drug Resistance TB treatment sites (PMDT). There are now 34 active PMDT sites to date. The treatments are provided through the national TB control programme, delivering free medication and social support to patients. All treatments provided to patients were in accordance with the WHO recommended DOTs strategy (2011 at the time). Patients typically visited their respective PMDT site every month for 24 months, which included routine follow-ups, laboratory testing, delivery of drugs and social support. A study conducted in Punjab, Pakistan demonstrated that advanced age and previous history of TB treatment were the primary cause of MDR-TB [9]. Several studies have shown different treatment success outcomes for patients with MDR-TB depending on the geographical region. For example, the success rate among the 2008–2013 cohort in Khyber Pakhtunkhwa (KPK) was between 63 and 79% [9–11], while studies from Rawalpindi identified a 10% success rate [9, 12] and Karachi had an approximate 39% success rate [9, 13]. Although TB treatment regimens are standardised and similarly practiced in different regions, success rates of MDR-TB treatment significantly vary between regions, where on average 65% of patients completed treatments. Studies are required to highlight confounding factors which may affect patient compliance, treatment outcomes and the quality of care contributing to these variations between regions.

It should be of highest priority to prevent MDR-TB by providing immediate access to high quality and effective drug treatments, expand rapid testing, prevent disease transmission through infection control measures and increase global involvement and funding [1]. The most important way to prevent future MDR-TB cases is through patient education and compliance. The Global fund to fight AIDs, malaria and TB, funded the National TB programme of Pakistan (NTP) to improve diagnostics and treatment, resource mobilisation, upgrade infrastructure, provide training and support research programmes [1]. With investments, the programme has achieved and sustained 70–94% treatment success rates in drug-susceptible TB patients while the success rate for MDR-TB was approximately 64% [1]. Despite these promising trends, treatment success for MDR-TB is persistent around 60–65% in Pakistan [1]. Despite increasing funding and growing efforts by NTP, MDR-TB imposes a great burden on the Pakistani population. Like other countries who are heavily affected by TB, Pakistan is a developing country facing many political and economic challenges. Political crises render it difficult for patients to seek medical care, while many public and private funded hospitals are also negatively impacted during peak crises [14]. Economic issues directly affect patient compliance and the quality of care provided by treatment sites. Due to economic strains, patients with TB may delay seeking medical care, especially since most low-income families do not have health insurance and medical expenses may cost more than their monthly allowance. Families with low-income tend to have larger families (8–10 people) that share small living spaces. A single member infected with TB in a large family unit may significantly increase transmission both within households and local communities [15]. In addition, a strong correlation between economic status and education level exists in Pakistan [16]. Limited education can hinder comprehension of disease and treatment information, leading to improper drug use and subsequently drug resistance. A study conducted in Islamabad, interviewed 36 patients from three different regions, at different stages of anti-TB treatment. The majority of patients were of poor socioeconomic status, had limited knowledge of TB and most were unwilling to disclose previous TB treatment [17].

In addition, extensive travelling to seek treatment is a major challenge for patients. Despite 34 active PMDT sites in Pakistan, patients are required to travel long distances to seek medical care. Long travel distances and associated costs may limit patients from seeking repeated treatments required for MDR-TB, potentially transitioning into extensive drug-resistance TB (XDR-TB). Travelling in general is more challenging for female patients, who have limited freedom and access to healthcare in Pakistan [18]. Studies suggest that the majority of patients spent up to three hours travelling, while spending an additional three to four hours at treatment centres. Patients primarily complained of fatigue and weakness during travelling but also mentioned frustration when drugs were not available at treatment facilities. Patients delayed or altogether avoided treatments due to long travelling times, costly trips and unavailability of drugs [17]. In addition, patients living in low-income communities have restricted access to social support programmes [19]. Social support programmes can not only educate patients but create supportive community culture. These programmes can improve awareness of TB, teach patients to identify TB-associated symptoms, seek medical care when required and ensure timely follow-ups [20]. These are a few socioeconomic and sociocultural factors which need to be considered when designing effective treatment plans for MDR-TB patients.

Identification of factors which prevent patient compliance, decrease accessibility to treatment and overall reduce favourable outcomes is required. This retrospective cohort study aims to highlight the demographics of all MDR-TB patients recorded by NTP between 2014 and 2017. Patient history, patient socioeconomic status and patient age were analysed with patient outcomes in Pakistan. This study also investigated the association between travelled distance to PMDT site and treatment outcomes in Sindh, Punjab and KPK. This study highlights important factors which need to be considered and implemented into current anti-TB treatment programmes. The establishment of new PMDT sites or community treatment sites along with funded transportation options can significantly improve patient compliance and treatment outcomes of MDR-TB patients.

## Methods

### Study design

**Fig. 1.**
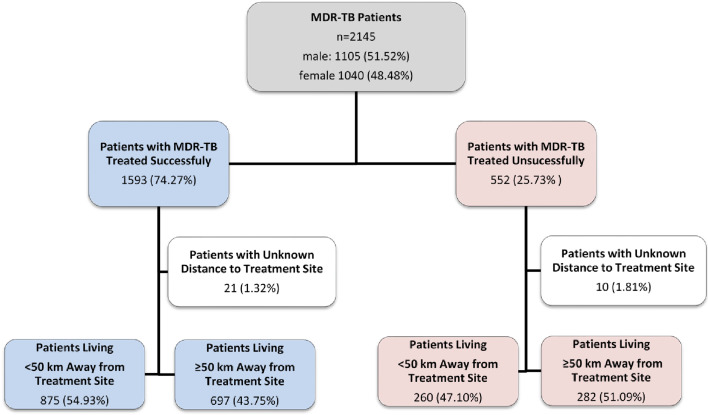
Study flow diagram of patients with MDR-TB, Pakistan, 2014–2017.

This study had a retrospective design and was a clinical record review of patients receiving MDR-TB at PMDT in Pakistan from January 2014 to December 2017 (Fig. 1).

### Setting

Pakistan is a developing country where the healthcare system is evolving with its expanding population. Communicable diseases, like TB, are the leading causes of both mortality and morbidity within the country. Both hospital and preventative care in rural and urban areas are financed primarily by the public sector. Factors like socioeconomic status, political and economic stability, housing and sanitation, insufficient healthcare infrastructure and population migration impact both MDR-TB rates and treatment.

In 2010, the Pakistani NTP began the pilot of PMDT in both ambulatory and hospital settings in three hospitals (National Tuberculosis Control Program Pakistan, 2014). At these PMDT sites, dedicated staff treated patients diagnosed with MDR-TB in line with the national guidelines. Patients were all given diagnostic services, socio-psychological support and treatment with SLDs. All drugs recommended by WHO were available in the country through the global drug facility, which were supported by the global fund. The usage of appropriate drugs was determined by drug-susceptibility testing and the patient's previous history with anti-TB drugs. The drugs used at that time included Amikacin/Capreomycin, Levofloxacin/Moxifloxacin, Cycloserine, Ethionamide, Para-aminosalicylic acid (if needed) and Pyrazinamide. Suspected MDR-TB patients were evaluated and diagnosed with a phenotypic drug-susceptibility test and the Xpert^®^ MTB/RIF assay (Cepheid, Sunnyvale, CA, USA). Patients who were subsequently diagnosed with MDR-TB were then offered care at the closest PMDT site to start treatment. At the PMDT sites, MDR-TB staff and physicians assessed and reviewed patients, their referral documents, medical history, laboratory and radiological test results and a physical exam was performed. After the completion of patient assessment, a treatment plan was created and entered into a treatment care card, followed by patient registration.

### Study population

Each PMDT site reported MDR-TB registered data on a routine reporting schedule monthly. The data set included patients who were cured or completed treatment and were categorised as having a favourable outcome and those who failed, died or did not complete treatment were categorised as having an unfavourable outcome. In our study, variables such as TB treatment history, age, sex, clinical information, type of resistance and treatment outcomes were collected from the MDR-TB register. Demographic information including socioeconomic status, were collected from patient files. One of the objectives was to investigate the impact of distance to treatment sites (in kilometers) on patient outcomes. MDR-TB data on treatment outcomes were collected from the same register. Data were collected from patients who received treatment at PMDT sites during January 2014 to December 2017. Geographical data were obtained from the database of global administrative areas (GADM) to map the distribution of favourable outcomes per province and states in Pakistan.

### Calculation of travelled distance

The data collected from patients included the region of patient's residence and the identified PMDT location for treatment. Using an online distance calculator (https://www.distancebetweencities.us/), approximate driving distance was calculated in kilometres.

### Data analysis

Verified and coded data were analysed using R (version 3.5.1; The R Foundation). Geographical data were mapped and analysed by ArcMap v.10.7.1. Descriptive analysis was undertaken and evaluated using cross-tabulation of demographic factors for all PMDT sites to compare between patients who were cured and not cured from MDR-TB. MDR-TB treatment was considered favourable if the patients were cured or if their treatment was completed without any indication of failure (Table 1). Demographic factors including nationality, age, province and socioeconomic status were all assessed. Patients were categorised into low, middle and high-level socioeconomic status based on general categorisation of recorded employment. Service jobs were mainly categorised as low-level occupation, while employment in government and public institutes were categorised as middle or high-level occupation. Mean, modes and standard deviations were calculated to assess and summarise the study population (Table 1). Stratification by potential confounding variables including nationality, age, gender, province, socioeconomic status and treatment history was undertaken. A chi-squared test was employed to test if there was a significant relationship between each categorical variable and having a favourable treatment outcome (Table 1). To assess the relationship between socioeconomic status and patient treatment outcomes, patients were grouped by socioeconomic status and further stratified by previous treatment outcomes. To assess the impact of treatment history and having a favourable treatment outcome per socioeconomic status, a chi-squared test was conducted (Table 2). Patients with unknown previous treatment outcomes were excluded from the chi-squared test. To analyse the impact of patient treatment history per province on treatment outcomes, patients were grouped into KPK, Sindh or Punjab. Patients were then stratified by their previous treatment history and a chi-squared test was undertaken to understand the relationship between treatment history and having a favourable treatment outcome per province (Table 3). Patients with unknown previous treatment outcomes were excluded from the chi-squared test. Logistic regression was performed for all provinces to explain the relationship between the binary variable, favourable or unfavourable outcome and the distance to PMDT sites. Both unadjusted and adjusted models were carried out to understand how covariates of interest influenced the relationship between distance to treatment sites and having a favourable or unfavourable treatment outcome. Subsequently, unadjusted regression models were performed for all provinces, KPK, Sindh and Punjab, to determine if there were any region-specific differences in the odds of having a favourable treatment outcome. Categorical variables were converted into dummy variables so that the odds for each sub-category could be calculated. Reference categories were determined for each covariate group based on the sub-category with the largest *n*-value. Only significant covariates were included in the final adjusted multivariate regression which were age and distance to treatment site. Adjusted odds ratios (ORs), 95% confidence intervals and *p*-values were calculated to assess the association between distance to treatment site and having a favourable treatment outcome. To assess the provincial differences for treatment outcomes, patients were categorised into KPK, Sindh or Punjab sub-groups (Table 4).

**Table 1.** Counts, Percentages and Differences in Demographic Factors for MDR-TB Patients With Favourable and Unfavourable Outcomes

	Overall	Favourable Outcome	Unfavourable Outcome	*p*-value
	(*n* = 2145)	(*n* = 1593)	(*n* = 552)	
Nationality				
Pakistan	2129 (99.25%)	1578 (99.06%)	551 (99.82%)	0.198
Afghanistan	15 (1.17%)	14 (0.88%)	1 (0.18%)	
Uzbekistan	1 (0.05%)	1 (0.06%)	0 (0.00%)	
Age*				
<25	727 (33.89%)	593 (37.23%)	132 (23.91%)	<0.001*
25-49	1071 (49.93%)	802 (50.34%)	271 (49.09%)	
>50	347 (16.18%)	198 (12.43%)	149 (26.99%)	
Gender				
Female	1040 (48.48%)	780 (48.96%)	260 (47.10%)	0.450
Male	1105 (51.52%)	813 (51.04%)	292 (52.90%)	
Province/State				
KPK	165 (7.69%)	130 (8.16%)	35 (6.34%)	0.328
Sindh	1025 (47.79%)	771 (48.40%)	254 (46.01%)	
AJK	7 (0.33%)	4 (0.25%)	3 (0.54%)	
Balochistan	88 (4.10%)	62 (3.89%)	26 (4.71%)	
FATA	29 (1.35%)	22 (1.38%)	7 (1.27%)	
Punjab	812 (37.86%)	587 (36.85%)	225 (40.76%)	
Gilgit Baltistan	1 (0.03%)	1 (0.06%)	0 (0.00%)	
Federal Capital Territory	18 (0.84%)	16 (1.00%)	2 (0.36%)	
Distance to Treatment Site (km)				
<50	1135 (53.69%)	875 (55.66%)	260 (47.97%)	0.012*
50–99	393 (18.59%)	281 (17.87%)	112(20.66%)	
100–149	270 (12.77%)	185 (11.77%)	85 (15.68%)	
150–199	145 (6.86%)	111 (7.06%)	34 (6.27%)	
>200 km	171 (8.09%)	120 (7.63%)	51 (9.41%)	
No Distance	31 (1.45%)	21 (67.74%)	10 (32.26%)	
Socioeconomic Status				
Unemployed	1151 (60.77%)	848 (60.61%)	303 (61.21%)	
Low	536 (28.30%)	400 (28.59%)	136 (27.47%)	
Middle	115 (9.99%)	82 (5.86%)	33 (6.67%)	
High	92 (4.86%)	69 (4.93%)	23 (4.65%)	
Not listed	251 (11.70%)	194 (13.87%)	57 (10.33%)	0.888
Treatment history				
New	169 (7.89%)	127 (7.98%)	42 (7.62%)	0.041*
Other (previously treated)	606 (28.29%)	439 (27.59%)	167 (30.31%)	
Previously treated (after failure)	759 (35.43%)	572 (35.95%)	187 (33.93%)	
Previously treated (after lost to follow up)	56 (2.61%)	36 (2.26%)	20 (3.62%)	
Previously treated (relapse)	299 (13.96%)	239 (15.02%)	60 (10.89%)	
Unknown previous treatment History	253 (11.81%)	178 (11.19%)	75 (13.61%)	
Not listed	3 (0.14%)	2 (0.13%)	1 (0.18%)	

Data are presented as *n* (%); *Indicates significance

**Table 2.** Effects of Socioeconomic Status on Patient Treatment Outcome

Socioeconomic Status	Favourable Outcomes (*n* = 1258)	Unfavourable Outcomes (*n* = 427)	*p*-value
Unemployed (*n* = 1019)	*n* = 757	*n* = 262	
New (*n* = 93)	67 (8.85%)	26 (9.92%)	0.158
Failure (*n* = 417)	302 (39.89%)	115 (43.89%)	
After lost to follow up (*n* = 28)	19 (2.51%)	9 (3.44%)	
Relapse (*n* = 161)	132 (17.44%)	29 (11.07%)	
Other (*n* = 320)	237 (31.31%)	83 (31.68%)	
Low (*n* = 481)	*n* = 363	*n* = 118	
New (*n* = 28)	20 (5.51%)	8 (6.78%)	0.343
Failure (*n* = 190)	149 (41.05%)	41 (34.75%)	
After lost to follow up (*n*=16)	9 (2.48%)	7 (5.93%)	
Relapse (*n* = 72)	55 (15.15%)	17 (14.41%)	
Other (*n* = 175)	130 (35.81%)	45 (38.14%)	
Middle (*n* = 100)	*n* = 74	*n* = 26	
New (*n* = 9)	7 (9.46%)	2 (7.69%)	0.011*
Failure (*n* = 40)	33 (44.59%)	7 (26.92%)	
After lost to follow up (*n* = 4)	1 (1.35%)	2 (11.54%)	
Relapse *(*n = 19)	17 (22.97%)	2 (7.69%)	
Other (*n* = 28)	16 (21.62%)	12 (46.15%)	
High (*n* = 85)	*n* = 64	*n* = 21	
New (*n* = 18)	15 (23.44%)	3 (14.29%)	0.647
Failure (*n* = 24)	16 (25.00%)	8 (38.10%)	
After lost to follow up (*n* = 2)	2 (3.13%)	0 (0.00%)	
Relapse (*n* = 15)	12 (18.75%)	3 (14.29%)	
Other (*n* = 26)	19 (29.69%)	7 (33.33%)	

Data are presented as *n* (%); *Indicates significance

**Table 3.** Effects of Patient Treatment History Per Province on Treatment Outcomes

Province	Favourable Outcomes Per Province	Unfavourable Outcomes Per Province	*p*-value
KPK (*n* = 145)	*n* = 115	*n* = 30	
New (*n* = 15)	14 (12.17%)	1 (3.33%)	0.037*
Failure (*n* = 62)	45 (39.13%)	17 (56.67%)	
After lost to follow up (*n* = 5)	2 (1.74%)	3 (10.00%)	
Relapse (*n* = 35)	29 (25.22%)	6 (20.00%)	
Other (*n* = 28)	25 (21.74%)	3 (10.00%)	
Sindh (*n* = 924)	*n* = 699	*n* = 225	
New (*n* = 80)	57 (8.15%)	23 (10.22%)	0.726
Failure (*n* = 392)	300 (42.92%)	92 (40.89%)	
After lost to follow up (*n* = 20)	15 (2.15%)	5 (2.22%)	
Relapse (*n* = 141)	111 (15.88%)	30 (13.33%)	
Other (*n* = 291)	216 (30.90%)	75 (33.33%)	
Punjab (*n* = 682)	*n* = 498	*n* = 184	
New (*n* = 62)	48 (9.64%)	14 (7.61%)	0.130
Failure (*n* = 247)	183 (36.75%)	64 (34.78%)	
After lost to follow up (*n* = 24)	15 (3.01%)	9 (4.89%)	
Relapse (*n* = 98)	79 (15.86%)	19 (10.33%)	
Other (*n* = 251)	173 (34.74%)	78 (42.39%)	

Data are presented as *n* (%); *Indicates significance

**Table 4.** Unadjusted and Adjusted Odds Ratios for 50 km Distance to Treatment Site Predicting Favourable/Unfavourable Outcomes of MDR-TB Patients

	ORs (95% CI) 1	*p*-values	aORs (95% CI)	*p*-values
All Provinces (*n* = 2114)				
Distance to treatment site (50 km)	0.95 (0.90–0.99)	0.010*	0.94 (0.90–0.99)	0.010*
Age	0.97 (0.96–0.98)	<0.001*	0.97 (0.96–0.98)	<0.001*
Gender				
Male	1.00			
Female	1.08 (0.89–1.31)	0.458		
Province				
KPK	1.22 (0.83–1.85)	0.322		
Sindh	1.00			
Azad Kashmir (AJK)	0.44 (0.10–2.24)	0.284		
Balochistan	0.79 (0.49–1.29)	0.324		
FATA	1.04 (0.46–2.64)	0.937		
Punjab	0.86 (0.70–1.06)	0.156		
ICT	2.64 (0.74–16.74)	0.198		
Socioeconomic Status				
Unemployed	1.00			
Low	1.05 (0.83–1.33)	0.671		
Middle	0.89 (0.59–1.37)	0.587		
High	1.07 (0.67–1.79)	0.777		
Treatment History				
New	0.99 (0.68–1.47)	0.953		
Other (previously treated)	0.86 (0.67–1.10)	0.222		
Previously treated (after, failure)	1.00			
Previously treated (after lost to, follow up)	0.59 (0.34–1.06)	0.069		
Previously treated (relapse)	1.30 (0.94–1.81)	0.120		
KPK (*n* = 165)				
Distance to Treatment Site (50 km)	0.72 (0.52–0.99)	0.046*	0.74 (0.53–1.02)	0.065
Age	0.98 (0.95–1.00)	0.061	0.98 (0.95–1.00)	0.077
Gender				
Male	1.00			
Female	1.24 (0.57–2.64)	0.578		
Socioeconomic Status				
Unemployed	1.00			
Low	0.95 (0.36–2.84)	0.925		
Middle	1.14 (0.16–22.92)	0.907		
High	1.14 (0.16–22.92)	0.907		
Treatment History				
New	5.29 (0.95–99.47)	0.121		
Other (previously treated)	3.15 (0.94–14.42)	0.089		
Previously treated (after, failure)	1.00			
Previously treated (after lost to follow up)	0.25 (0.03–1.64)	0.149		
Previously treated (relapse)	1.83 (0.67–5.55)	0.257		
Sindh (*n* = 1025)				
Distance to Treatment Site (50 km)	0.93 (0.87–0.99)	0.037*	0.93 (0.87–0.99)	0.032*
Age	0.97 (0.96–0.98)	<0.001*	0.97 (0.96–0.98)	<0.001*
Gender				
Male	1.00			
Female	1.18 (0.88–1.57)	0.265		
Socioeconomic status				
Unemployed	1.00			
Low	0.95 (0.68–1.33)	0.769		
Middle	0.75 (0.39–1.54)	0.413		
High	1.31 (0.61–3.12)	0.515		
Treatment History				
New	0.76 (0.45–1.32)	0.317		
Other (previously treated)	0.88 (0.62–1.26)	0.489		
Previously treated (after failure)	1.00			
Previously treated (after lost to follow up)	0.92 (0.35–2.89)	0.875		
Previously treated (relapse)	1.13 (0.72–1.83)	0.595		
Punjab (n = 812)				
Distance to Treatment Site (50 km)	0.95 (0.87–1.03)	0.172	0.94 (0.87–1.02)	0.153
Age	0.97 (0.96–0.98)	<0.001*	0.97 (0.96–0.98)	<0.001*
Gender				
Male	1.00			
Female	1.03 (0.76–1.41)	0.831		
Socioeconomic status				
Unemployed	1.00			
Low	1.10 (0.76–1.61)	0.615		
Middle	0.93 (0.53–1.68)	0.802		
High	0.92 (0.47–1.86)	0.799		
Treatment History New	1.20 (0.63–2.39)	0.590		
Other (previously treated)	0.78 (0.52–1.15)	0.202		
Previously treated (after, failure) Previously treated (after lost to, follow up) Previously treated (relapse)	1.00 0.58 (0.25–1.45) 1.45 (0.83–2.64)	0.226 0.203		

*Indicates significance; - indicates reference group; Gilgit Baltistan excluded (*n* = 1)

### Ethics approval

This study obtained ethics exemption and authorisation to use data from the Pakistan NTP. Due to the retrospective nature of this study, direct patient contact and informed consent was not required.

## Results

### Demographics of MDR-TB patients in Pakistan (2014–2017)

Patients were stratified based on demographic factors, distance travelled to PMDT sites and treatment history to identify any differences in treatment outcomes. Of the 2145 patients who received MDR-TB treatment from January 2014 to December 2017, most patients were of Pakistani nationality (99.3%) (Table 1). The average age of patients was 33.4 years. There were slightly more males who received treatment during the study time period. The highest proportion of treated patients were registered in Sindh (47.7%). Most patients (53.7%) travelled less than 25 km to a PMDT site. In terms of socioeconomic status, 60.8% of MDR-TB patients were unemployed at the time of the treatment. When assessing previous treatment history, 35.4% of MDR-TB patients had previously received treatment after failure.

**Fig. 2.**
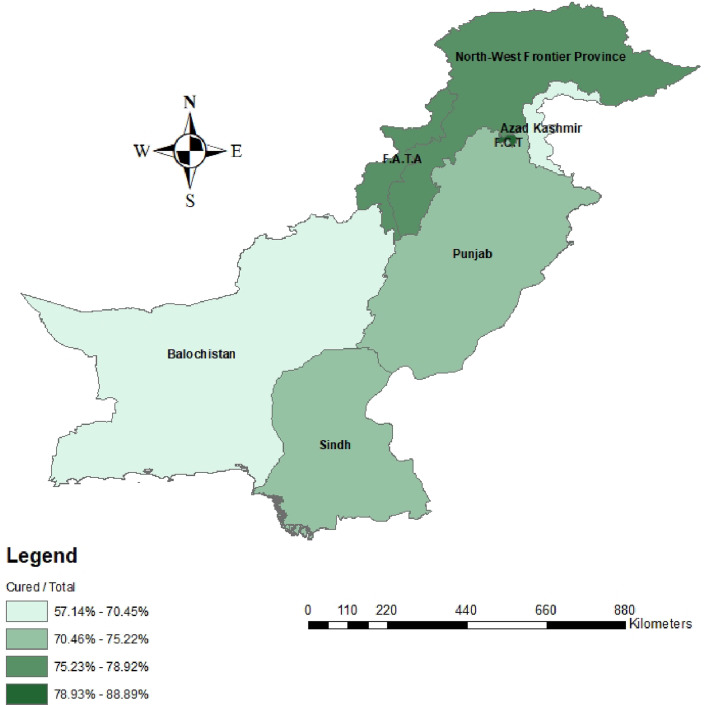
Choropleth map representing favorable patient outcomes based on Pakistan’s provinces and states.

When comparing between favourable and unfavourable outcomes, patients were once again stratified by demographic factors and treatment history to identify differences in treatment outcomes (Table 1). Nationality did not have an impact on having a favourable outcome following MDR-TB treatment. Age was significantly associated with being cured or completing treatment (*P* < 0.001). Gender did not influence having a favourable treatment outcome. When comparing provinces, there were no significant differences in treatment outcomes for any of the provinces. Majority of patients travelled less than 50 km and the distance travelled to treatment sites was significantly associated with treatment outcomes (*P* = 0.01). When comparing patient socioeconomic status, there were no significant differences observed between any of the strata. Lastly, significant differences in patient outcomes were observed between various patient treatment histories (*P* < 0.05). To summarise, a four-quantile choropleth map demonstrated favourable outcomes that were normalised based on the total population per province in Pakistan. More favourable outcomes were observed in northern Pakistan, including the North-West Frontier Province (KPK) and Federally Administered Tribal Area (FATA) (79–89%) compared to Balochistan (57–70%), Sindh and Punjab (70–75%) (Fig. 2).

### Patients who were unemployed or were of middle-level socioeconomic status and had previously relapsed or failed treatment were more likely to have favourable outcomes, respectively

Patient socioeconomic status and previous treatment history were stratified to assess differences in treatment outcomes (Table 2). Overall, unemployment was not a significant demographic factor in assessing treatment outcomes after stratifying by previous treatment history. Although, unemployed patients who relapsed after previous treatment were more likely to be cured (17.4% *vs.* 11%). Patients with low-level socioeconomic status with varying treatment histories had non-significant differences in having a favourable outcome. There was a significant relationship between having a middle-level occupation and patient's having a favourable treatment outcome (*P* = 0.01). Patients with middle-level occupations who had previously failed treatment, were more likely to have favourable treatment outcomes (44.6% *vs.* 26.9%). In addition, patients with middle-level occupations who were previously lost to follow up were less likely to be cured (1.4% *vs.* 11.5%). Lastly, patients with high-level occupations had no significant differences between previous treatment histories and current MDR-TB treatment outcomes.

### Differences in patient treatment histories affect treatment outcomes in Sindh, Punjab and KPK

Patients registered in Sindh, Punjab and KPK were stratified based on previous treatment history. As expected, most registered MDR-TB patients had previously failed treatment (Table 3). In Sindh, approximately 42.9% of patients with favourable outcomes previously failed therapy, 30.9% were categorised as other, 15.9% had previously relapsed and 8.2% were new cases in relation to total cured cases in Sindh. In Punjab, approximately 36.8% of patients with favourable outcomes previously failed treatment, 34.7% were categorised as other, 15.9% had previously relapsed and 9.6% were new patients in relation to total cured cases in Punjab. In KPK, approximately 39.1% of patients with favourable outcomes previously failed treatment, 25.2% had previously relapsed, 21.7% categorised as other and 12.2% of patients were newly registered in relation to total cured cases in KPK. There was a significant relationship between previous treatment history in KPK and patient's having a favourable treatment outcome (*P* = 0.037). The greatest percentage of patients with unfavourable outcomes (56.7%, 40.9%, 34.8%) were registered as ‘previous failed treatment’ in KPK/Sindh and ‘other’ in Punjab, respectively.

### Travelled distances to PMDT sites significantly impact patient treatment outcomes

Approximate distances travelled by patients to PMDT sites were calculated and were significantly associated with patient outcomes for all provinces (Table 1). In our logistic regression model, distance to treatment site (50 km) was significantly associated with treatment outcomes in all provinces (OR 0.95, 95%CI 0.90–0.99) (Table 4). Age was the only other significant predictor of participants having a favourable outcome (OR 0.97, 95%CI 0.96–0.98). All other potential covariates, including gender, province, socioeconomic status and treatment history were not significant predictors when analysed independently to predict favourable treatment outcomes (Table 4). In KPK, distance to treatment sites was the only variable that was significantly associated with treatment outcomes (OR 0.72, 95%CI 0.52–0.99). There was a trend for increasing age to reduce the odds of having a favourable treatment outcome (OR 0.98, 95%CI 0.95–1.00) (Table 4). In the adjusted logistic regression for KPK distance to treatment site and age were marginally not significant (aOR 0.74, 95%CI 0.53–1.02 and aOR 0.98, 95%CI 0.95–1.00, respectively). For Sindh, the distance to treatment site and age were significantly independent predictors of patient treatment outcomes. There was a 7% reduction in the odds of participants having a favourable treatment outcome for every 50 km increase in distance away from the treatment site (OR 0.93, 95%CI 0.87–0.99). For Sindh, there was a 3% reduction in the odds of patients having a favourable treatment outcome for every one-year increase in age (OR 0.97, 95%CI 0.96–0.98) (Table 4). Lastly, in the province of Punjab, the distance to treatment sites was not significantly predictive of treatment outcomes (OR 0.95, 95%CI 0.87–1.03). Although, the only predictor variable that was significant was age (OR 0.97, 95%CI 0.96–0.98).

In our adjusted logistic regression model, the distance to treatment site (km) and the covariate, age, were included. The results from the adjusted model suggest that with every 50 km increase in distance there is a 6% lower odds of patients being cured/completing treatment (aOR 0.94 *P* *=* 0.010*), while controlling for age. In addition, age was significantly associated with patient outcomes (aORs 0.97, 95%CI 0.96–0.98) (Table 4).

Thus, in the adjusted multivariate regression model both distance to treatment site and age were included. In the final adjusted model neither distance to treatment site nor age remained significant for the province of KPK (aOR 0.74 *P* *=* 0.065; aOR 0.98 *P* = 0.077, respectively). In the adjusted regression model, distance to treatment site still remained a significant predictor after adjusting for participant's age in the province of Sindh (aOR 0.93, *P* *=* 0.03*2*) and age remained a significant predictor after adjusting for distance to treatment site (aOR 0.97, *P* < 0.001). In the final adjusted model for Punjab, distance to treatment sites remained insignificant while age was a significant predictor (aOR 0.97, 95%CI 0.96–0.98).

## Discussion

From January 2014 to December 2017, 2145 patients were documented to begin and complete MDR-TB treatment, where most patients (74.2%) had favourable treatment outcomes and 25.7% of patients were not cured of MDR-TB (Fig. 1). Of the demographic, clinical and treatment history factors that were assessed; age, socioeconomic status and previous treatment relapse had an association with MDR-TB treatment outcomes. Patients younger than 25 were more likely to be cured. However, even in this age group 23.91% of patients were not cured, indicating room for improvement in treatment accessibility. Patients over 50 were less likely to have favourable treatment outcomes (12.4% *vs.* 27.0%) (Table 1). Ncube *et al*. (2017) found that the elderly had worse treatment outcomes compared to younger cohorts of patients, which is congruent with our study [21]. This study also indicated that worse outcomes may be due to other immunosuppressant conditions and age-related diseases [21]. This is evident that other comorbid conditions may create unique challenges for the treatment of older MDR-TB patients. These coexisting conditions could increase TB patients’ risk of adverse drug reactions, recurrence rates and mortality [22–24].

The majority of patients registered in Sindh, Punjab and KPK, travelled less than 50 km to seek treatment at the nearest PMDT site. The distance travelled to PMDT sites significantly impacted patient outcomes in all provinces (Table 1). In all provinces, patients who travelled less than 50 km had greater favourable outcomes than any other distance group. In all provinces, the risk of an unfavourable outcome increased for patients who travelled greater than 50 km, when compared to patients who travelled less than 50 km. When separating patients by province, most patients travelled less than 50 km in KPK, Sindh and Punjab but a large portion of patients travelled longer than 100 km in each province (Supplementary Table 1). Overall, patients who travelled longer than 50 km had less favourable outcomes when stratified based on individual province.

The presented logistic regression model demonstrated a significant association that for every 50 km increase in travel distance, there would be 7.0% lower odds of being cured after adjusting for the potential covariate age (Table 4). Similarly, many published studies identified distance to TB treatment sites as a primary factor regarding patient compliance and treatment completion [25–27]. A study completed in Islamabad, interviewed patients to identify factors which prevented access to TB treatment [17]. The majority of patients travelled an average of three hours to PMDT sites. Patients disclosed that they delayed, or all together avoided trips to PMDT sites due to long travel times. Patients also identified expensive costs for long distance travelling combined with physical fatigue as reasons for interrupting treatment. In addition, female patients identified long travel distances, associated costs and inability to travel alone contributed to non-compliance [17]. In our study, approximately 48% of registered patients were female, therefore it is important to consider individual population needs when developing treatment strategies. To improve patient compliance and prevent inappropriate use of TB medicine, WHO developed the directly observed treatment short-course (DOTs) strategy to prevent the spread of TB in communities with high incidence rates [28]. With the implementation of the DOTs strategy, researchers have published success rates up to 94%, matching WHO criteria [29, 30]. Despite the effectiveness of this strategy, patients are still faced with many challenges limiting access to treatment. Maheshwary *et al*. (2017) identified limited access to PMDT sites and inadequate patient education as primary factors associated with low patient compliance [31]. Consequently, the DOTs strategy needs to be refined to overcome these challenges and increase the number of registered patients at PMDT sites. A potential strategy to improve accessibility is to establish funded bus routes in communities that are located further from PMDT sites. Funding provided from WHO and/or the Pakistani government can implement transportation, which can also include travel vouchers, incentives or taxi fares to mobilise patients from their communities to the nearest PMDT site. Mixed methods studies identified financial burden associated with transportation as an important factor for patient compliance [15, 32]. Majority of patients had a monthly income of less than 2000 rupees, where roughly 100–200 rupees were spent on travel [15,32]. Many patients needed to be accompanied to clinics, further adding on the financial strain [15, 32]. Funded and scheduled transportation routes can significantly reduce the financial burden on patients and increase the number of patients actively seeking medical treatment. Interviewed patients also identified fatigue and weakness during long travel. Patients who are weak are also less receptive to anti-TB therapy [33]. Providing lunch for patients during travelling can also encourage patients to seek treatment while ensuring healthier patients. Scheduled transportation can also benefit treatment facilities. With known arrival of patients, facilities can ensure appropriate quantities of medication, hospital beds and other resources to accommodate patients scheduled for treatment. This can prevent long delays for both hospital staff and patients while also reducing the risk of unavailable treatments. In addition, a community-based approach may further decrease the distance between patients and medical facilities. Employing community-level healthcare providers can further encourage patients to seek medical care especially within their familiar community [25, 34]. The distribution of favourable patient outcomes by geographical locations, highlighted provinces that need special attention regarding accessibility and improvements in patient care (Fig. 2). Future planning strategies are required to modify current approaches in specific provinces including Balochistan and Azad Jammu and Kashmir (AJK). The total population of both provinces is approximately 16 million people, which is roughly one-third of the population of Sindh. Compared to the number of patients registered in Sindh (*n* = 1025), only 95 patients were registered cumulatively, and 66 patients completed treatment (Table 1). It is possible that either fewer patients in these regions have MDR-TB or that fewer patients are actually being registered and treated for MDR-TB. Limited resources in Balochistan and accessibility limitations in Azad Kashmir due to higher elevations and Himalayan regions can hinder access to and availability of treatment. The implementation of satellite clinics or telemedicine may provide immediate patient consults and online treatment instructions. Although the DOTs strategy is effective in treating TB patients, refinements and modifications including bus transportation, or community-based approaches may significantly increase favourable outcomes of MDR-TB patients.

Treatment outcomes between PMDT sites were also analysed and compared (Supplementary Table 2). Although no retrospective study comparing all PMDT sites has been conducted to date, a systematic review was conducted in 2018 investigating TB management by different healthcare practitioners in Pakistan [35]. This review identified variabilities in TB diagnosis, standard of care and practitioner knowledge between provinces in Pakistan. In certain provinces, knowledge and use of sputum microscopy was poor, many practitioners did not use smear microscopy for diagnosis and many practitioners demonstrated limited knowledge of diverse TB-associated symptoms [35]. This review identified a ‘know-do gap’ highlighting a gap between what practitioners know and their actual practice [35]. Another study identified the lack of resources and equipped facilities in public sector facilities. Only 48% of public hospitals had access to microscopy services. Approximately 50% of public hospitals in Punjab and only 23% in Sindh had microscopy services [36]. Currently, of the 34 operating PMDT sites in Pakistan, 31 of those facilities are part of the public sector. It is important to note that all levels of healthcare facilities were included in our study. Based on these data, more work is required to strengthen the quality of both diagnosis and treatment in the public sector. In our present study, PMDT sites with the most favourable patient outcomes (78%) in Sindh and Punjab were from the private sector (Indus Hospital Karachi (IHK) and Gulab Devi Chest Hospital (GDH)) (Supplementary Table 2). It is likely that private funding ensures better access to advanced diagnostic and treatment regimens, encouraging practitioners to close the gap between their knowledge and actual practice. It is also important to note that public hospitals which specialise in chest diseases, including Ojha Institute of Chest Diseases (OICD), Institute of Chest Diseases Kotri (ICDK) and Gulab Devi Chest Hospital (GDH) had favourable patient outcomes, ranging between 73 and 78%. It is likely that hospitals which specialise in chest diseases are likely to have better diagnostic tools and protocols in place. To better diagnose and treat MDR-TB patients, Pakistan needs to steer away from an individualistic approach and implement a more collaborative strategy. Collaborations between hospitals which specialise in chest diseases, teaching hospitals and better equipped facilities may further improve patient management. Transfer of knowledge between treatment facilities will be the fastest method to increase the number of cured patients to the target established by WHO. In addition, private−public sector partnerships and collaborations can further improve patient management and allocation of resources.

Previous history of anti-TB treatment is the major risk factor for the onset of MDR-TB. Many patients with MDR-TB have previous history with improper use of first-line anti-TB drugs [6, 37]. A study conducted at OICD in 2009, demonstrated that all enrolled patients (*n* = 579) had a previous history with multiple courses of first-line anti-TB drugs. The success rate was approximately 39% in these patients, where supervised treatment was provided to all patients [13]. In our study, the majority of patients had previously failed treatment in Sindh, KPK and Punjab. It is unclear whether failed therapy included patients who stopped taking anti-TB drugs or completed treatment but failed to treat TB. In either case, approximately 37–43% of favourable cured patients had previously failed treatment (Table 3). There were no drastic differences in favourable or unfavourable outcomes when patients who were previously treated either after failure or relapse, suggesting the need for prioritising these patients (Table 1). Support from family and healthcare practitioners can improve patient compliance. Self-motivation, knowledge of TB and treatment, counselling, family support and nutritional support can influence patients to adhere to anti-MDR-TB treatment [38]. Shifting from a one-treatment-for-all strategy towards a patient-centred approach including personalised treatment plans, counselling, nutritional supplementation and social support may significantly increase drug compliance and prevention of MDR-TB [38]. It is critical to implement patient-centred strategies immediately for new patients to prevent future failure or relapse to TB.

Sharma *et al*. (2019) assessed factors associated with developing MDR-TB and found that middle-aged patients (between 30 and 50 years) were more likely to develop MDR-TB which was similarly observed in our study [39]. Majority of MDR-TB patients were between the ages of 25 and 49 (49.9%). Other studies conducted in Hong Kong, Ethiopia and Bangladesh reported that middle-aged individuals were more likely to be diagnosed with MDR-TB, indicating the unique age-related challenges faced by adults in Pakistan diagnosed with the disease [25,40,41]. In our study, patients older than 50 were less likely to be cured of MDR-TB. Mobility challenges or transportation issues in older patients may lead to difficulties in accessing PMDT sites. This identifies unique factors contributing to patient outcomes and emphasises the need for modification of the current programme in order to accommodate older adults and improve accessibility to PMDT sites. A potential solution is by offering subsidized or complimentary transportation to older adults who may live further away from PMDT sites.

This is the largest study investigating treatment outcomes of MDR-TB patients in Pakistan. This study is the first to compare patient outcomes between different provinces in Pakistan. In addition, this study is the first to look at the association between socioeconomic status and previous MDR-TB treatment history. This information is critical to identifying patients of different working classes who may have poor previous treatment adherence leading to unfavourable treatment outcomes. This the first study to identify differences in patient outcomes between PMDT sites in Sindh and Punjab. This information is critical for the NTP to support PMDT sites which have limited relative resources, by providing improved resources, initiating inter-facility collaborations or by implementing advanced training for practitioners. Lastly and more importantly, this is the first study to show significant implications on patient outcomes in relation to travel distances. These results are important for the NTP to modify national strategies that target specific patient-related challenges. Establishment of funded scheduled bus routes can reduce the financial burden on patients who are required to travel long distances and ensure patients receive scheduled anti-TB treatments.

Limitations of this study include its retrospective nature and the use of preexisting patient information collected by NTP. This is the largest database of MDR patients in Pakistan but included missing patient information. Missing patient information encompassed gaps in patient medical history, living conditions, employment and exact location of residence. Due to these limitations, the exact distance travelled could not be calculated but approximate distances were calculated between the district of residence and PMDT sites. The method utilised for calculating distance quantified the total distance travelled and did not include the duration or types of transportation. In addition, many patients were documented as ‘unknown’ regarding patient treatment history. These ambiguous groups may have affected statistical analysis regarding patient history and patient outcomes. A future prospective study is required with a larger study population, which can identify clustering of patients with unfavourable treatment outcomes who lived further away from specific PMDT sites and individual districts. This will help develop site and district specific strategies to improve patient accessibility and close the gap between treatment delivery. In addition, correlations of drug treatment regimens and patient outcomes is required to determine optimal and suboptimal MDR-TB treatment regimens contributing to unfavourable treatment outcomes.

## Conclusions

Our study investigated various demographic variables associated with treatment outcomes for patients diagnosed with MDR-TB. Age was significantly associated with treatment outcomes, as younger patients (<25) were more likely to be cured compared to patients older than 50. Patients with middle-level occupations were more likely to be cured compared to patients with lower and higher-level occupations when accounting for previous treatment history. Patients with a previous history of anti-TB treatment had favourable treatment outcomes, since approximately 70–80% of patients were cured following re-treatment. Distance between patients and PMDT sites was a significant contributing factor to patient treatment outcomes. Most patients who travelled less than 50 km were significantly more likely to be cured *vs.* patients who travelled further than 100 km. With every 50 km increase in distance travelled, there was a 7.0% lower odds of a favourable treatment outcome while adjusting for potential confounding factors. Our study identified key factors including decentralisation of PMDT sites to improve treatment access for patients, establishment of strong collaborations with PMDT sites that specialise in chest diseases, increase support and funding for food and travel, all of which can be targeted and improved by the NTP to increase favourable outcomes of MDR-TB patients in Pakistan.

## Data Availability

The raw data that support the findings of this study can be accessed with the permission of the National Tuberculosis Project of Pakistan (Dr Hussain Hadi: Hussain.hadi@ntp.gov.pk).

## Supplementary material

For supplementary material accompanying this paper visit http://dx.doi.org/10.1017/S0950268820002307. click here to view supplementary material
