# Supplementary material for: Understanding how geographic, demographic and treatment history impact health outcomes of patients with multi-drug-resistant tuberculosis in Pakistan, 2014–2017
Source: Epidemiol Infect. 2020 Sep 30;148:e253. doi: 10.1017/S0950268820002307 (PMC7689600; doi:10.1017/S0950268820002307)
Supplement: Supplementary file 1 [file S0950268820002307sup001.docx]

Supplementary Table 1. Differences between treatment outcomes based on distance to PMDT site for Sindh, Punjab and KPK			
**Distance to Treatment Site**	**Favorable Outcomes Per Total Patients in Province (n=1475)**	**Unfavorable Outcome Per Total Patients in Province (n=513)**	**p-value**
KPK* (n=153)	***n=118***	***n=35***	
<50 km (n=95)	80 (67.80%)	15 (42.86%)	0.008*
50-99 km (n=25)	17 (14.40%)	8 (22.86%)	0.236
100-149 km (n=25)	14 (11.86%)	11 (31.43%)	0.006*
150-199 km (n=7)	6 (5.08%)	1 (2.86%)	0.582
200< km (n=1)	1 (0.84%)	0 (0.00%)	0.588
Sindh (1025)	***n=771***	***n=254***	
<50 km (n=533)	414 (53.70%)	119 (46.85%)	0.058*
50-99 km (n=205)	152 (19.71%)	53 (20.87%)	0.689
100-149 km (n=141)	103 (13.36%)	38 (14.96%)	0.521
150-199 km (n=80)	59 (7.65%)	21 (8.27%)	0.750
200< km (n=66)	43 (5.58%)	23 (9.06%)	0.050*
Punjab (n=810)	***(n=586)***	***(n=224)***	
<50 km (n=453)	337 (57.50%)	116 (51.78%)	0.143
50-99 km (n=157)	108 (18.43%)	49 (21.87%)	0.268
100-149 km (n=74)	48 (8.19%)	26 (11.60%)	0.132
150-199 km (n=53)	41 (6.99%)	12 (5.35%)	0.399
200< km (n=73)	52 (8.87%)	21 (9.37%)	0.824

Data is presented as n (%); * Indicates significance

Supplementary Table 2. Comparing Cured and Not Cured outcomes for PMDT Treatment Sites in Sindh and Punjab			
**Treatment Site**	**Favorable Outcomes Per PMDT site**	**Unfavorable Outcomes Per PMDT site**	**p-value**
**Sindh (n=1017)**	**(n= 765)**	**(n= 252)**	
CMC (n=69)	52 (75.36%)	17 (24.64%)	NS
GMMCH (n=162)	117 (72.22%)	45 (27.78%)	NS
ICDK (n=319)	243 (76.18%)	76 (23.82%)	NS
IHK (n=127)	100 (78.74%)	27 (21.26%)	NS
JPMC (n=51)	38 (74.51%)	13 (25.49%)	NS
MPK (n=105)	80 (76.19%)	25 (23.81%)	NS
OICD (n=146)	107 (73.29%)	39 (26.71%)	NS
PMCH (n=38)	28 (73.29%)	10 (26.32%)	NS
**Punjab (n=786)**	**(n=568)**	**(n=218)**	
BVH (n=32)	23 (71.88%)	9 (28.13%)	NS
FSD (n=2)	2 (100.00%)	0 (0.00%)	NS
GDH (n=124)	97 (78.23%)	27 (21.77%)	0.107
JHL (n=77)	55 (71.43%)	22 (28.57%)	NS
MHL (n=98)	66 (67.43%)	32 (32.65%)	NS
NHM (n=184)	133 (72.28%)	51 (27.72%)	NS
RLH (n=89)	62 (69.66%)	27 (30.34	NS
SARG (n=83)	61 (73.49%)	22 (26.51%)	NS
SKT (n=6)	4 (66.67%)	2 (33.33%)	NS
SSM (n=51)	39 (76.47%)	12 (23.53%)	NS
SZH (n=40)	26 (65.00%)	14 (35.00%)	NS

Data is presented as n (%); * Indicates significance
